# Biosynthesis of Poly(3-hydroxybutyrate-*co*-3-hydroxyhexanoate) From Glucose by *Escherichia coli* Through Butyryl-CoA Formation Driven by Ccr-Emd Combination

**DOI:** 10.3389/fbioe.2022.888973

**Published:** 2022-05-12

**Authors:** Shu Saito, Ryu Imai, Yuki Miyahara, Mari Nakagawa, Izumi Orita, Takeharu Tsuge, Toshiaki Fukui

**Affiliations:** ^1^ School of Life Science and Technology, Tokyo Institute of Technology, Yokohama, Japan; ^2^ School of Materials and Chemical Technology, Tokyo Institute of Technology, Yokohama, Japan

**Keywords:** metabolic engineering, poly(3-hydroxybutyrate-*co*-3-hydroxyhexanoate), polyhydroxyalkanoates, *Escherichia coli*, reverse *β*-oxidation

## Abstract

Poly[(*R*)-3-hydroxybutyrate-*co*-(*R*)-3-hydroxyhexanoate] [P(3HB-*co*-3HHx)] is a practical kind of bacterial polyhydroxyalkanoates (PHAs). A previous study has established an artificial pathway for the biosynthesis of P(3HB-*co*-3HHx) from structurally unrelated sugars in *Ralstonia eutropha*, in which crotonyl-CoA carboxylase/reductase (Ccr) and ethylmalonyl-CoA decarboxylase (Emd) are a key combination for generation of butyryl-CoA and the following chain elongation. This study focused on the installation of the artificial pathway into *Escherichia coli*. The recombinant strain of *E. coli* JM109 harboring 11 heterologous genes including Ccr and Emd produced P(3HB-*co*-3HHx) composed of 14 mol% 3HHx with 41 wt% of dry cellular weight from glucose. Further investigations revealed that the C_6_ monomer (*R*)-3HHx-CoA was not supplied by (*R*)-specific reduction of 3-oxohexanoyl-CoA but by (*R*)-specific hydration of 2-hexenoyl-CoA formed through reverse β-oxidation after the elongation from C_4_ to C_6_. While contribution of the reverse β-oxidation to the conversion of the C_4_ intermediates was very limited, crotonyl-CoA, a precursor of butyryl-CoA, was generated by dehydration of (*R*)-3HB-CoA. Several modifications previously reported for enhancement of bioproduction in *E. coli* were examined for the copolyester synthesis. Elimination of the global regulator Cra or PdhR as well as the block of acetate formation resulted in poor PHA synthesis. The strain lacking RNase G accumulated more PHA but with almost no 3HHx unit. Introduction of the phosphite oxidation system for regeneration of NADPH led to copolyester synthesis with the higher cellular content and higher 3HHx composition by two-stage cultivation with phosphite than those in the absence of phosphite.

## Introduction

Bacterial polyhydroxyalkanoates (PHAs) are eco-friendly polymeric materials that can be produced from renewable biomass resources and show high biodegradability (Taguchi et al., 2012; Guzik et al., 2020; and Sen and Baidurah, 2021). In particular, recently, the high biodegradability of PHAs in marine environments is drawing much attention as one of the promising solutions against marine pollution by plastic wastes and microplastics (Narancic et al., 2018; Waring et al., 2018). Poly[(*R*)-3-hydroxybutyrate-*co*-(*R*)-3-hydroxyhexanoate] [P(3HB-*co*-3HHx)] is a practical kind of PHA copolymer. It shows a lower melting temperature and crystallinity than poly[(*R*)-3-hydroxybutyrate] [P(3HB)] homopolymer, attributed to the long side chain in the 3-hydroxyhexanoate (3HHx) unit (Doi et al., 1995). The copolyesters are composed of about 5–15 mol% 3HHx exhibiting flexible properties suitable for several applications (Doi et al., 1995; Taguchi et al., 2012).

P(3HB-*co*-3HHx) is usually produced from vegetable oils and fatty acids by some wild strains such as *Aeromonas* spp. having PHA synthase with uniquely broad substrate specificity to (*R*)-3-hydroxyacyl-CoAs of C_4_–C_6_ (Doi et al., 1995; Han et al., 2004). In *Aeromonas caviae*, the C_6_ monomer (*R*)-3HHx-CoA is provided through the channeling of β-oxidation from 2-hexenoyl-CoA by the function of (*R*)-specific enoyl-CoA hydratase PhaJ (Fukui et al., 1998). Previous studies focused on P(3HB-*co*-3HHx) biosynthesis from vegetable oils or fatty acids by recombinant bacterial strains, and the introduction of *phaJ* was an important modification for generation of (*R*)-3HHx-CoA *via* β-oxidation (Fukui et al., 1999; Mifune et al., 2010; and Budde et al., 2011). In addition to oils and fatty acids, structurally unrelated sugars should also be considered for P(3HB-*co*-3HHx) biosynthesis as another way for the low-cost production. It is notable that no wild microbe capable of synthesizing the copolyester composed of high 3HHx fraction from sugars has been isolated so far. While, recent metabolic engineering has allowed to construct recombinant strains for biosynthesis of various PHAs from structurally unrelated carbon sources. *Ralstonia eutropha*, of which wild strain H16 has been known to be an efficient producer of P(3HB) homopolymer, has been engineered for P(3HB-*co*-3HHx) biosynthesis from fructose (Fukui et al., 2002; Insomphun et al., 2015). This was achieved by the design and introduction of an artificial pathway for building the (*R*)-3HHx-CoA monomer from three acetyl-CoA molecules. The key reaction is formation of butyryl-CoA from crotonyl-CoA by the combination of NADPH-dependent crotonyl-CoA carboxylase/reductase derived from *Methylorubrum extorquens* (Ccr_*Me*_) and ethylmalonyl-CoA decarboxylase from mammalian *Mus musculus* (Emd_*Mm*_). The resulting butyryl-CoA was then elongated to C_6_ intermediates by condensation with the third acetyl-CoA and subsequently converted to (*R*)-3HHx-CoA. When this artificial pathway was installed into an *R. eutropha* strain having PhaC_NSDG_ (a mutant PHA synthase derived from *A. caviae*) and lacking PhaB1 (the major NADPH-acetoacetyl-CoA reductase), the strain produced P(3HB-*co*-22.2 mol% 3HHx) with 48 wt% content of the dry cell weight from fructose (Insomphun et al., 2015). Zhang et al (2019) integrated the engineering for the copolyester synthesis with modifications for glucose assimilation and enhancement of reverse β-oxidation in *R. eutropha*, resulting in production of P(3HB-*co*-12.1 mol% 3HHx) with 75 wt% cellular content from glucose.

Non-PHA-producing *Escherichia coli* has also been frequently used as the host for metabolic engineering aiming at PHA production owing to its fast growth and availability of versatile genetic tools (Li et al., 2007). The lack of an intracellular depolymerization system is also considered an advantage for PHA production. Highly efficient production of P(3HB) (157 g/L) has been reported by glucose-fed batch cultivation of recombinant *E. coli* harboring *phaCAB1* from *R. eutropha* along with filamentation suppression by overexpression of *ftsZ* (Wang and Lee, 1997). Wang et al (2015) reported biosynthesis of P(3HB-*co*-13.2 mol% 3HHx) from glucose by recombinant *E. coli* with 12.9 wt% cellular content through a pathway including reverse β-oxidation, in which butyryl-CoA formation was mediated by NADH-dependent *trans*-enoyl-CoA reductase (Ter) from *Treponema denticola*. In this study, the pathway containing butyryl-CoA formation driven by the combination of Ccr and Emd and the following reverse β-oxidation was introduced into *E. coli* for P(3HB-*co*-3HHx) synthesis from glucose.

## Materials and Methods

### Bacterial Strains and Plasmids

The strains and plasmids used in this study are listed in Supplementary Table S1. *Escherichia coli* strains DH5α and JM109 were routinely cultivated at 30°C or 37°C in a lysogeny broth (LB) medium composed of 1% (w/v) tryptone (Nacalai Tesque, Kyoto, Japan), 0.5% (w/v) yeast extract (Becton Dickinson, Franklin Lakes, NJ, United States), and 1% (w/v) NaCl. Carbenicillin (100 μg/ml), kanamycin (100 μg/ml), gentamycin (30 μg/ml), and/or chloramphenicol (30 μg/ml) were added into the medium when necessary.

### Plasmid Construction

DNA manipulations were carried out according to standard procedures, and PCR reactions were performed with KOD-Plus ver.2 DNA polymerase (Toyobo, Osaka, Japan). The sequences of oligonucleotide primers used in this study are shown in Supplementary Table S2. The transformation of *E. coli* strains were performed according to conventional chemical competent or electroporation procedures.

pBKS-PCJAB was constructed by inserting a KpnI-XbaI restriction fragment of pBBR1*phaP*(D4N)*CJ*_*Ac*_ *AB*_*Re*_ (Ushimaru et al., 2015) containing *P*_*Ac*_ *-phaP*_D4N_ *C*_NSDG_ *J*_*Ac*_ and *P*_*Re*_-*phaAB1*_*Re*_ into pBluescript II KS(+) at the corresponding sites, where *P*_*Ac*_ and *P*_*Re*_ are native promoter regions of *phaPCJ* from *A. caviae* and *phaCAB1* from *R. eutropha*, respectively. pBKS-PCJA was obtained by deleting the *phaB1*_*Re*_ region from pBKS-PCJAB by inverse PCR followed by self-ligation. pBtac-CJ_Re_E was constructed by replacing the *phaP* promoter region in pBPP-ccr_Me_J4a-emd (Insomphun et al., 2015) by the *tac* promoter region amplified from pBBRtac (Fukui et al., 2011). pBtac-CJ_Re_EB and pBtac-CJ4_Pa_E were derivatives of pBtac-CJ_Re_E obtained by insertion of *bktB*_*Re*_ at downstream of *emd*_*Mm*_ and replacement of *phaJ4a*_*Re*_ by *phaJ4*_*Pa*_, respectively. pSTV-HC was constructed by replacement of the *lac* promoter-*lacZα* region in pSTV28 by a tandem of *had*_*Re*_ and *crt2*_*Re*_ fused with a *tac* promoter. Further insertion of *bktB*_*Re*_ into pSTV-HC at the downstream of *crt2*_*Re*_ gave pSTV-HCB. pSTV-PCB was constructed by replacement of *had*_*Re*_ in pSTV-HCB by *paaH1*_*Re*_. pMW-Gm-pxtD_EAAR_ABC for phosphite dehydrogenation was constructed by inserting an EcoRI-SacI restriction fragment containing *pxtD*_EAAR_ *ABC* excised from pBBR1MCS2::*pxtD*_EAAR_ *ABC* (Miyahara et al., 2018) into pMW218-Gm which is a pMW218 derivative harboring Gm^r^ instead of Km^r^.

### Construction of *E. coli* Knockout Strains

*E. coli* single-gene knockout mutants and the parent strain BW25113 in the Keio collection (Yamamoto et al., 2009) were obtained from the National BioResource Project (National Institute of Genetics, Japan): *E. coli*. The *Km* ^*r*^ gene inserted within the target gene was removed by using temperature-sensitive pCP20 harboring *flp* as described previously (Cherepanov and Wackernagel, 1995). Double-gene knockout strains JW∆cra∆rng, JW∆pgi∆rng, and JW∆pta∆poxB were constructed by additional deletion of the second gene (*rng* or *poxB*) from the corresponding single deletion strains by using λ-Red recombinase-mediated recombination (Datsenko and Wanner, 2000). In the case of JW∆pta∆poxB for an example, the FLP recombinase target (FRT)-Km^r^-FRT region flanked to 50-bp extensions in *poxB* was amplified from the genomic DNA of JW∆poxB by PCR. JW∆pta/pKD46 grown in the presence of 0.1% l-arabinose for the expression of λ-Red recombinase was transformed with the corresponding PCR product by electroporation. The cells were incubated in an SOC medium at 37°C for 1 h and inoculated onto an LB plate medium containing 50 μg/ml kanamycin. The colonies formed on the Km-LB plate medium were picked, and ampicillin sensitivity of the clones was confirmed in the LB medium with 50 μg/ml ampicillin. Insertion of the Km^r^ cassette at the target locus in the obtained clones was confirmed by PCR, and the cassette was then removed by using pCP20.

### Production and Analyses of PHA

*E. coli* strains were cultivated at 30°C in 100 ml of LB medium supplemented with 20 g/L glucose. Appropriate antibiotics were added at the final concentration as described previously, and 1.0 mM IPTG was added into the medium for induction of gene expression prior to inoculation. After the cultivation for 48 h with reciprocal shaking (115 strokes/min), the cells were harvested, washed once with cold deionized water, and then lyophilized. The cellular PHA content and composition were determined by gas chromatography (GC) after direct methanolysis of the dried cells in the presence of 15% sulfuric acid as described previously (Kato et al., 1996).

PHA polymers were extracted from the dried cells by stirring in chloroform for 72 h at room temperature and recovered by precipitation with methanol. The molecular weight and polydispersity were determined by gel permeation chromatography (GPC) using a Shimadzu 10A GPC system and a 10A refractive index detector equipped with a serial of Shodex K-806M and K-802 columns at 40°C. Chloroform was used as the eluent at a flow rate of 0.8 ml/min. The calibration curve was generated using polystyrene standards with a low polydispersity (STANDARD SM-105, Shodex, Tokyo, Japan).

### Substrate and Metabolite Analyses

Glucose, acetate, and pyruvate in the culture supernatant were measured using a flow injection analyzer BF-9 (Oji Scientific Instruments, Hyogo, Japan) at 25°C. Glucose concentration was determined using a glucose oxidase-based biosensor with a mobile phase composed of 100 mM Na_3_PO_4_, 50 mM KCl, and 1 mM NaN_3_ (pH 7). Acetate and pyruvate were simultaneously measured by using a serial of pyruvate kinase- and acetokinase/pyruvate kinase/pyruvate oxidase-based biosensors, respectively, and 50 μM FAD, 50 μM thiamine pyrophosphate, 0.2 mM ATP, and 0.2 mM phosphoenolpyruvate were added to the mobile phase.

Phosphite and phosphate concentrations were determined by a capillary electrophoresis P/ACE system MDQ (Beckman Coulter, CA, United States) and anion analysis kit at 25°C. The analytes were separated with 20 kV voltage detected by the indirect detection mode. The capillary was washed with 0.1 N NaOH and 0.1 N HCl before analysis, and the samples were injected by vacuum (0.5 psi for 8.0 s) (Miyahara et al., 2018).

## Results

### Pathway Design and Plasmid Construction

**FIGURE 1:**
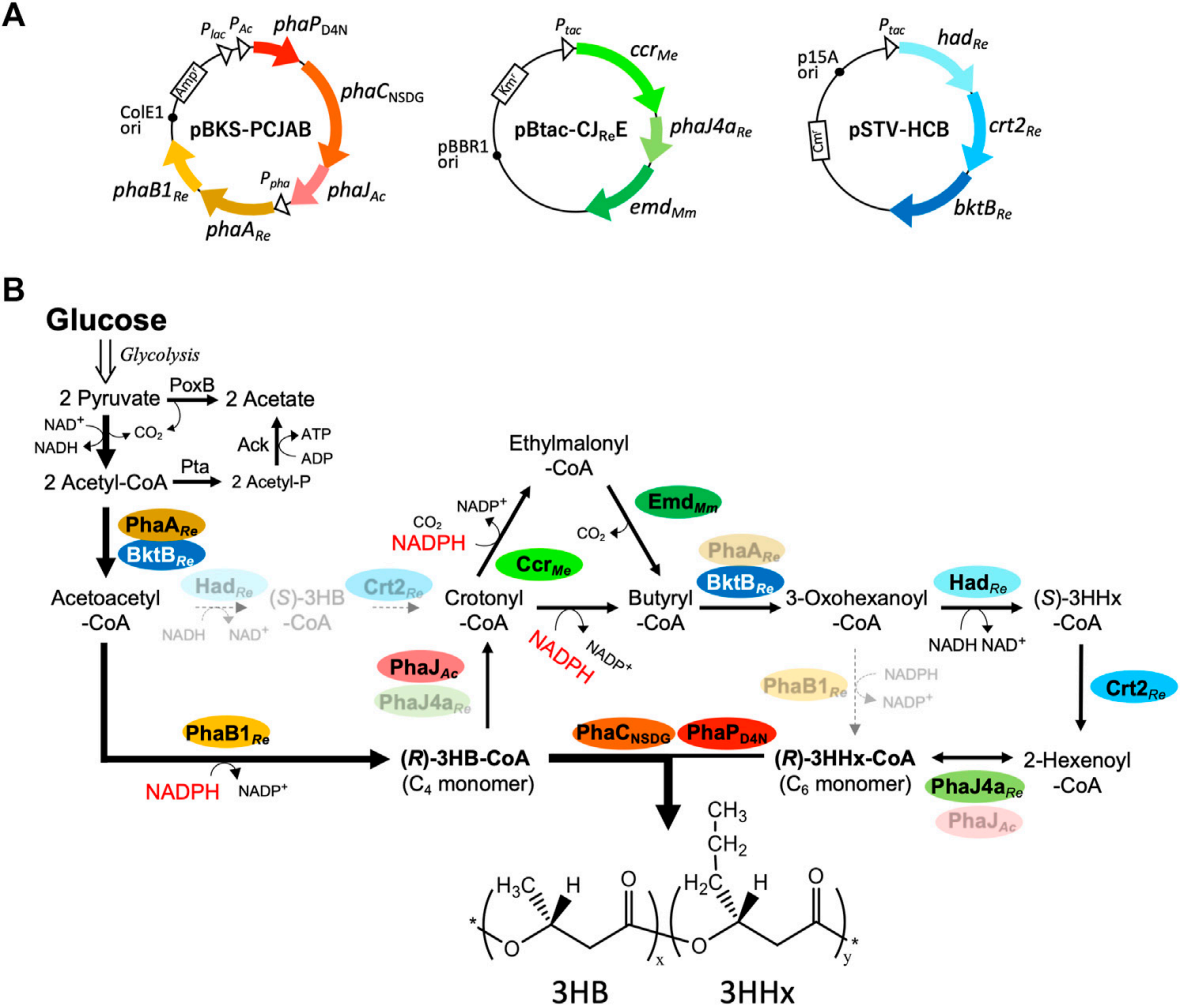
Plasmids **(A)** and artificial pathways **(B)** introduced into *E. coli* for biosynthesis of P(3HB-*co*-3HHx) from glucose. PhaP_D4N_, D4N mutant of phasin from *A. caviae;* PhaC_NSDG_, N149S/D171G mutant of PHA synthase from *A. caviae*; PhaJ and PhaJ4a, (*R*)-enoyl-CoA hydratases; PhaA and BktB, β-ketothiolases; Ccr, crotonyl-CoA carboxylase/reductase; Emd, ethylmalonyl-CoA decarboxylase (codon-optimized); Had, NAD-(*S*)-3HB-CoA dehydrogenase, Crt2, crotonase; Pta, phosphate acetyltransferase; Ack, acetate kinase; PoxB, pyruvate oxidase. *Ac*, *Aeromonas caviae*; *Re*, *Ralstonia eutropha*; *Me*, *Methylorubrum extorquens*; *Mm*, *Mus musculus*.

The pathway for the biosynthesis of P(3HB-*co*-3HHx) from glucose introduced into *E. coli* is shown in Figure 1. The C_4_ monomer (*R*)-3HB-CoA is conventionally formed through two reaction steps: condensation of two molecules of acetyl-CoA and subsequent reduction with (*R*)-stereospecificity to (*R*)-3HB-CoA. Two kinds of β-ketothiolase [PhaA_*Re*_ and BktB_*Re*_ (Slater et al., 1998)] and NADPH-acetoacetyl-CoA reductase (PhaB1_*Re*_) derived from *R. eutropha* are applied for these two reactions in this study. Two pathways for crotonyl-CoA generation from acetoacetyl-CoA are dehydration of (*R*)-3HB-CoA catalyzed by (*R*)-specific enoyl-CoA hydratase from *A. caviae* (PhaJ_*Ac*_) (Fukui et al., 1998; Mifune et al., 2010) and (*S*)-specific pathway mediated by NAD^+^-(*S*)-3-hydroxyacyl-CoA dehydrogenase and crotonase [(*S*)-specific enoyl-CoA hydratase] from *R. eutropha* (Had_*Re*_ and Crt2_*Re*_, respectively) (Segawa et al., 2019). Crotonyl-CoA is then converted to butyryl-CoA or ethylmalonyl-CoA by bifunctional Ccr_*Me*_ catalyzing NADPH-dependent reduction or reductive carboxylation, respectively (Erb et al., 2007). The latter is decarboxylated to butyryl-CoA by Emd_*Mm*_ (Linster et al., 2011), thus consequently converting crotonyl-CoA to butyryl-CoA by the combination of Ccr_*Me*_ and Emd_*Mm*_. Because the aforementioned BktB_*Re*_, Had_*Re*_, and Crt2_*Re*_ showed rather broad substrate specificity accepting C_4_–C_6_ intermediates, the three enzymes are expected to establish reverse β-oxidation for butyryl-CoA to 2-hexenoyl-CoA. A medium-chain-length-specific (*R*)-enoyl-CoA hydratase from *R. eutropha* (PhaJ4a_*Re*_) (Kawashima et al., 2012) then generates (*R*)-3HHx-CoA from 2-hexenoyl-CoA. (*R*)-3-Hydroxyacyl-CoAs of C_4_ and C_6_ are copolymerized by PhaC_NSDG_ (Asn149Ser/Asp171Gly double mutant of PHA synthase from *A. caviae*) (Fukui and Doi, 1997; Tsuge et al., 2007), which has been shown to synthesize P(3HB-*co*-3HHx) with a higher 3HHx composition than the wild-type enzyme.

These genes of the heterologous enzymes were introduced into *E. coli* by three compatible plasmids. pBKS-PCJAB harbored *phaP*_D4N_-*phaC*_NSDG_-*phaJ*_*Ac*_ and *phaA*_*Re*_ *-phaB1*_*Re*_, both with the native promoter regions derived from *A. caviae* and *R. eutropha*, respectively. *phaP*_D4N_ is a gene of Asp4Asn (D4N) mutant of PhaP (granule-associated protein, phasin), in which the nucleotide change corresponding to the D4N mutation has been reported to elevate expression levels of the *phaP-C-J* gene cluster in *E. coli* (Ushimaru et al., 2015). The *lac* promoter in the plasmid was also expected to contribute to the high expression of *phaP-C-J* potentially important for the efficient synthesis of PHAs. pSTV-HCB harbors a tandem of *had*_*Re*_, *crt2*_*Re*_, and *bktB*_*Re*_ located downstream of *P*_*tac*_. pBtac-CJ_Re_E was constructed by insertion of a tandem of *ccr*_*Me*_, *phaJ4a*_*Re*_, and codon-optimized *emd*_*Mm*_ into pBBRtac, a broad-host range expression plasmid containing *P*_*tac*_ (Fukui et al., 2011).

### P(3HB-*co*-3HHx) Biosynthesis From Glucose by the Engineered *E. coli*

**FIGURE 2:**
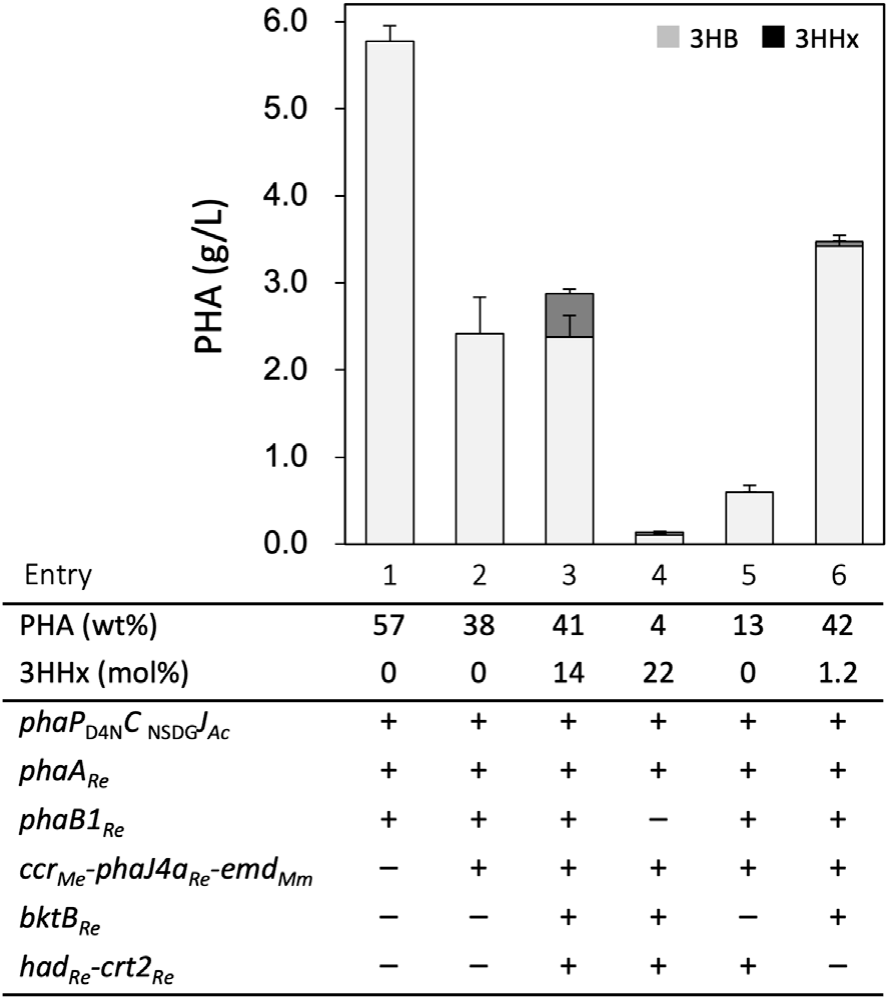
P(3HB-*co*-3HHx) biosynthesis from glucose by *E. coli* JM109-derived recombinant strains. The amounts of 3HB and 3HHx units in PHA are shown in gray and black bars, respectively. The cells were cultivated in a 100 ml LB medium containing 2% (w/v) glucose and 1 mM IPTG for 48 h at 30°C.

*E. coli* JM109 strains were transformed by the constructed plasmids and cultivated for PHA production at 30°C in the LB medium containing 2% (w/v) glucose with IPTG induction. The strain harboring *phaP*_D4N_ *C*_NSDG_ *J*_*Ac*_ and *phaAB*_*Re*_ on a high-copy number plasmid (pBKS-PCJAB) accumulated a large amount (5.6 g/L) of P(3HB) homopolymer with 57 wt% of the cellular dry weight (entry 1, Figure 2 and Supplementary Table S3). As this PHA production was higher than the previous case using a medium-copy number plasmid [pBBR1*phaPCJ*_*Ac*_AB_Re_ (Ushimaru et al., 2015)], pBKS-PCJAB was used as the module for (*R*)-3HB-CoA formation and polymerization for further engineering. Unlike in *R. eutropha* (Insomphun et al., 2015; Zhang et al., 2019), introduction of genes for butyryl-CoA formation (*ccr*_*Me*_-*phaJ4a*_*Re*_-*emd*_*Mm*_) did not enable the P(3HB)-producing *E. coli* to synthesize P(3HB-*co*-3HHx) from glucose, as the transformant having pBKS-PCJAB and pBtac-CJ_Re_E still produced P(3HB) homopolymer with a low content of 38 wt% (entry 2). P(3HB-*co*-3HHx) biosynthesis from glucose was finally achieved by further introduction of the third plasmid pSTV-HCB harboring the genes for reverse β-oxidation (*had*_*Re*_ *-crt2*_*Re*_ *-bktB*_*Re*_). This triple transformant produced 2.8 g/L of P(3HB-*co*-14 mol% 3HHx) with 41 wt% content after 48 h cultivation (entry 3). The incorporation of the 3HHx unit into PHA was not observed when the cultivation temperature was elevated up to 37°C, probably due to the unstable property of any of the heterologous enzymes for formation of (*R*)-3HHx-CoA monomer. All the cultivation was thus carried out at 30°C in this study.

**FIGURE 3:**
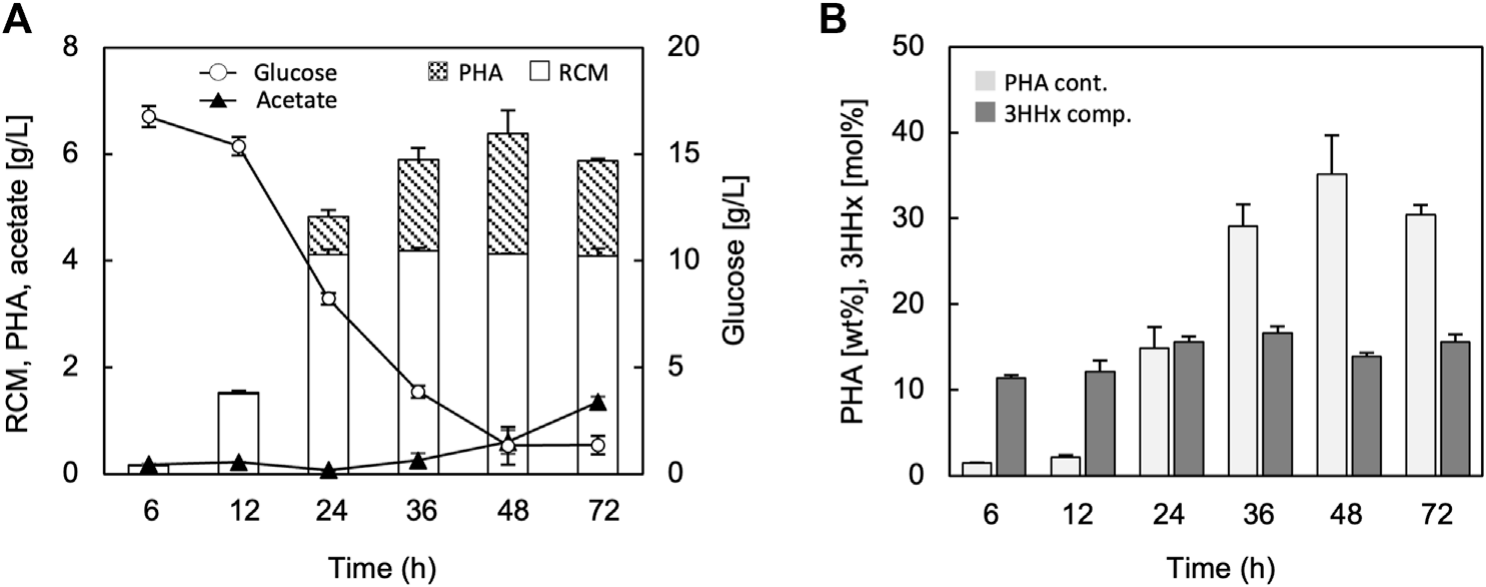
Time-dependent changes in the residual cell mass (RCM), PHA, glucose, and acetate **(A)**, and PHA content and 3HHx composition **(B)** through P(3HB-*co*-3HHx) biosynthesis from glucose by *E. coli* JM109 harboring pBKS-PCJAB/pBtac-CJ_Re_E/pSTV-HCB. The cells were cultivated in a 100 ml LB medium containing 2% (w/v) glucose and 1 mM IPTG for 48 h at 30°C.

The time-course of growth and PHA synthesis by the strain JM109/pBKS-PCJAB/pBtac-CJ_Re_E/pSTV-HCB are shown in Figure 3. The cell growth (residual cell mass, RCM) was saturated at 24 h (Figure 3A), and PHA accumulation was started from 12 h and continued to 48 h (Figures 3A,B). Although the PHA accumulation was looked to be decreased after 48 h, the difference of PHA amounts between 48 and 72 h was not significant (*p*-value > 0.25). The 3HHx composition was constant ranging within 12–14 mol% regardless of the cultivation time.

The number average-molecular weight (*M*_*n*_) and polydispersity (PDI) of P(3HB-*co*-14 mol%) synthesized by JM109/pBKS-PCJAB/pBtac-CJ_Re_E/pSTV-HCB were determined to be 5.80 ×10^4^ and 1.55, respectively, by GPC (Table 1). The molecular weight values were roughly half of P(3HB) homopolymer synthesized by the strain solely having pBKS-PCJAB, which would be due to some effect of incorporation of the C_6_ units on the enzymatic polymerization. Both the PHAs synthesized by the recombinant *E. coli* in this study showed a lower molecular weight but narrow distribution than P(3HB-*co*-12 mol% 3HHx) synthesized by the previously engineered *R. eutropha* strain from glucose (Zhang et al., 2019) (*M*_*n*_ and PDI were 29.5 ×10^4^ and 3.59, respectively). It has been reported that introduction of an additional copy of *phaC* resulted in the decrease in PHA molecular weight in *R. eutropha* (Kawashima et al., 2015). This agreed with the lower molecular weight in *E. coli* harboring *phaC* on the high-copy number plasmid than the previous *R. eutropha* strain having the single *phaC* gene, probably attributed to more active centers in the catalytic polymerization.

**TABLE 1:** Molecular weights of P(3HB-*co*-3HHx) synthesized by recombinant strains of *E. coli* and *R. eutropha* from glucose.

PHA	Producer	*M*_*n*_ (x10^5^)	*M*_*w*_ (x10^5^)	*M*_*w*_ */M*_*n*_
P(3HB)	*E. coli* JM109/pBKS-PCJAB	1.04 ± 0.21	2.23 ± 0.76	2.08 ± 0.27
P(3HB-*co*-14 mol% 3HHx)	*E. coli* JM109/pBKS-PCJAB/pBtac-CJ_Re_E/pSTV-HCB	0.580 ± 0.01	0.901 ± 0.01	1.55 ± 0.02
P(3HB-*co*-12 mol% 3HHx)^a^	*R. eutropha* NSDG-GG-HC/pBPP-ccr_Me_J_Ac_-Emd^a^	2.95 ± 1.01	10.6 ± 3.62	3.59 ± 0.01

a Zhang et al (2019).

### Identification of Pathways for (*R*)-3HA-CoA Formation

The roles of PhaB1 and reverse β-oxidation on the formation of (*R*)-3HA-CoAs of C_4_ and C_6_ in the recombinant *E. coli* were investigated by gene deletion analyses. The introduction of pBKS-PCJA lacking *phaB1*_*Re*_ resulted in a significant decrease in the accumulated polyester (entry 4). Although the 3HHx composition of 22 mol% was higher than that by the strain having *phaB1*_*Re*_, the amounts of 3HB and 3HHx units in the polymer fraction were remarkably reduced. This indicated the critical role of PhaB1_*Re*_ in the provision of both the monomers within the cells. When pSTV-HC-lacking *bktB*_*Re*_ was used, the strain synthesized P(3HB) homopolymer with low content (entry 5). The strain lacking *had*_*Re*_ and *crt2*_*Re*_ but harboring *bktB*_*Re*_ was constructed by double transformation with pBKS-PCJAB and pBtac-CJ_Re_EB, in which the latter plasmid contained *bktB*_*Re*_ at downstream of *emd*_*Mm*_. The resulting strain synthesized PHA containing only a trace fraction of the 3HHx unit although the accumulation was as much as that by the full triple transformant (entry 6), indicating the essential roles of the (*S*)-specific enzymes for formation of the C_6_-monomer.

It has been clarified that two kinds of (*S*)-3HB-CoA dehydrogenases Had and PaaH1 were active in *R. eutropha* grown on fructose or soybean oil (Segawa et al., 2019). Both the dehydrogenases have broad substrate specificity toward 3-oxoacyl-CoAs of C_4_–C_8_, in which PaaH1 showed slightly higher catalytic efficiency to the C_6_ substrate than Had, while one of the (*R*)-enoyl-CoA hydratases from *Pseudomonas aeruginosa* (PhaJ4_*Pa*_) was reported to show activity to *trans*-2-enoyl-CoAs of C_6_ and longer but no activity to crotonyl-CoA (Tsuge et al., 2003), different from PhaJ4a_*Re*_ showing low activity to crotonyl-CoA (Kawashima et al., 2012). We here replaced *had*_*Re*_ and *phaJ4a*_*Re*_ in the plasmids by *paaH1*_*Re*_ and *phaJ4*_*Pa*_, respectively. As shown in Supplementary Table S4, the strains having PaaH1 and/or PhaJ4_*Pa*_ produced P(3HB-*co*-3HHx) with similar cellular content and composition when compared to the parent strain having Had_*Re*_ and PhaJ4a_*Re*_. The catalytic properties of (*S*)-3HB-dehydrogenase and (*R*)-enoyl-CoA hydratase were not significantly affected by the PHA copolymer biosynthesis in *E. coli*.

### Effects of Mutation(s) on Sugar Metabolism-Regulating Genes and Acetate Formation

**FIGURE 4:**
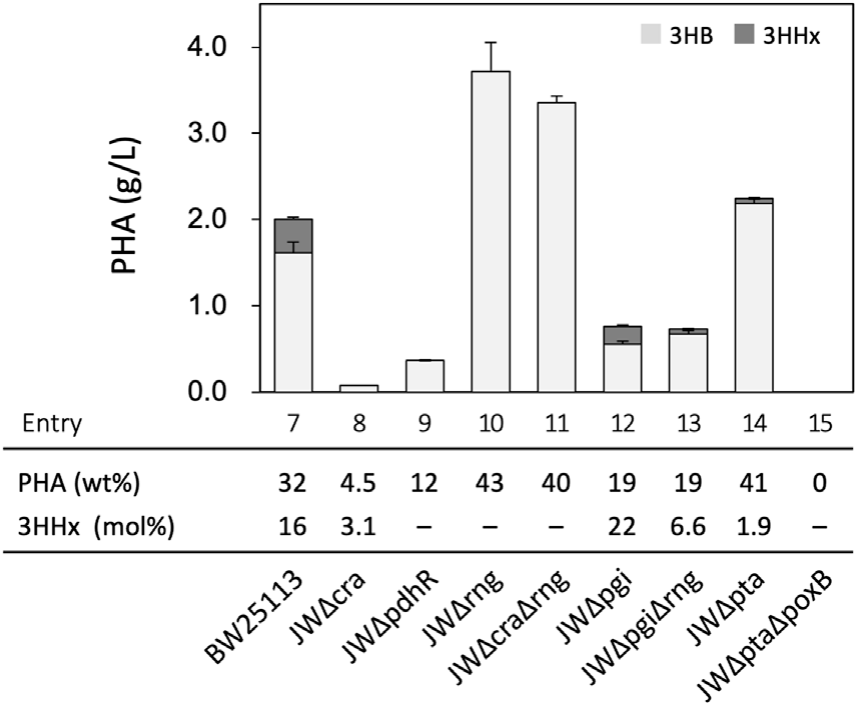
Effects of mutation(s) on sugar metabolism-regulating genes and acetate formation on P(3HB-*co*-3HHx) biosynthesis from glucose by *E. coli* BW25113-derived recombinant strains harboring pBKS-PCJAB/pBtac-CJ_Re_E/pSTV-HCB. The amounts of 3HB and 3HHx units in PHA are shown in gray and black bars, respectively. The cells were cultivated in a 100 ml LB medium containing 2% (w/v) glucose and 1 mM IPTG for 48 h at 30°C.

Further modifications were introduced based on previous knowledge for central metabolisms in *E. coli*. Several *E. coli* mutant strains were obtained from the Keio collection (Yamamoto et al., 2009) and used after the excision of kanamycin-resistant marker genes. The mutant strains transformed with pBKS-PCJAB/pBtac-CJ_Re_E/pSTV-HCB were subjected to PHA synthesis from glucose. *E. coli* BW25113 (a parent of Keio collection strains) harboring the three plasmids produced P(3HB-*co*-16 mol% 3HHx) with the cellular content of 32 wt% (entry 7, Figure 4 and Supplementary Table S5), whose property was similar to that of the JM109-based strain. Cra (FruR) is a global transcriptional regulator modulating central metabolisms and respiration (Ramseier et al., 1995; Saier and Ramseier, 1996), and PdhR is a pyruvate-sensing transcriptional repressor for genes of pyruvate dehydrogenase complex and respiration (Gohler et al., 2011; Maeda et al., 2017). Although we assumed that acetyl-CoA formation following PHA synthesis may be enhanced by deletion of *cra* (entry 8) or *pdhR* (entry 9), these modifications resulted in a marked decrease in both cell growth and PHA production. Acetate was formed by the JW∆cra-based strain in higher levels (35 mM) than the parent strain (Supplementary Table S5). RNase G, encoded by *rng*, is an endonuclease-cleaving rRNA precursor and glycolysis gene-derived mRNA. It was reported that pyruvate was overproduced by ∆*rng*∆*cra* double mutant of *E. coli* MG1655 possibly owing to enhanced glycolysis (Sakai et al., 2007). However, we here observed that the strain lacking *rng* produced PHA with a high cellular content (43 wt%) whereas only a trace amount of the 3HHx unit was detected in the polymer fraction (entry 10). The strain doubly lacking *rng* and *cra* (entry 11) showed the PHA biosynthesis property similar to that of the single ∆*rng* strain. Inactivation of phosphoglucose isomerase Pgi has been demonstrated to shunt glucose metabolisms from the Embden–Meyerhof pathway to NADPH-forming Entner–Doudroff and pentose-phosphate pathways, which was expected to be favorable for PHA synthesis since PhaB1 and Ccr_*Me*_ utilize NADPH as a reduced cofactor. The strain lacking *pgi* produced P(3HB-*co*-3HHx) with a high 3HHx composition (22 mol%), while the cellular PHA content was decreased to 19 wt% (entry 12). It was considered that double deletion of *pgi* and *rng* might compensate the respective disadvantages to each other; however unexpectedly, the JW∆pgi∆rng-based transformant produced P(3HB-*co*-6.6 mol% 3HHx) with a low cellular content of 19 wt% (entry 13).

We further investigated the effects of block of acetate formation (Dittrich et al., 2005) on the copolyester biosynthesis. The JW∆pta-based triple transformant lacking phosphate acetyltransferase Pta produced P(3HB-*co*-3HHx) with a high cellular content but low 3HHx composition (41 wt% and 1.9 mol% 3HHx, respectively) (entry 14) and still secreted acetate (Supplementary Table S5). The acetate formation was completely lost by double deletion of *pta* and *poxB* encoding pyruvate oxidase (entry 15). However, pyruvate was secreted instead of acetate, and both the cell growth and PHA production were severely impaired.

### Introduction of the Phosphite Oxidation System for NADPH Supply

Miyahara et al (2018) reported that NADPH supply coupled with phosphite oxidation led to a 3.2-fold increase in P(3HB) production in recombinant *E. coli* under non-growth conditions. We thus investigated the effects of the phosphite oxidation system on P(3HB-*co*-3HHx) biosynthesis by *E. coli*. The fourth plasmid pMW-Gm-ptxD_EAAR_ABC was constructed, in which *ptxD*_EAAR_ and *ptxABC* are genes encoding the Glu175Ala/Ala176Arg double mutant of phosphite dehydrogenase and phosphite transporters, respectively, both derived from *Pseudomonas stutzeri*. It has been reported that PtxD was NAD^+^-specific, but the mutant enzyme could accept NADP^+^ as well as NAD^+^ as the electron acceptor (Woodyer et al., 2003). The quadruple transformant of *E. coli* JM109 was subjected to two-stage cultivation on glucose in a MOPS-based mineral medium. The transformant having *ptxD*_EAAR_ *ABC* produced P(3HB-*co*-11 mol% 3HHx) with a cellular content of 31 wt% from glucose in the absence of phosphite, while the cellular content and 3HHx composition were significantly increased up to 48 wt% and 24 mol%, respectively, by addition of 5 g/l phosphite into the medium (Table 2).

**TABLE 2:** Effects of the phosphite oxidation system on P(3HB-*co*-3HHx) biosynthesis from glucose by two-stage cultivation of *E. coli* JM109 harboring pBKS-PCJAB/pBtac-CJ_Re_E/pSTV-HCB/pMW-Gm-ptxD_EAAR_ABC.

Phosphite (g/L)	Dry cell mass (g/L)	PHA (g/L)	Residual cell mass (g/L)	PHA content (wt%)	3HHx composition (mol%)	Phosphite consumption (g/L)	Glucose consumption (g/L)	Acetate formation (g/L)
0	1.46 ± 0.09	0.46 ± 0.11	1.00 ± 0.04	31.0 ± 6.0	11.0 ± 0.4	–	6.9 ± 0.3	0.37 ± 0.04
5	1.72 ± 0.02	0.82 ± 0.02	0.90 ± 0.02	48.0 ± 1.3	23.6 ± 0.3	1.0 ± 0.1	6.5 ± 0.1	0.77 ± 0.01

The cells grown in a 100-ml LB medium for 15 h at 30°C were harvested and then transferred to the MOPS-buffered mineral medium (40 mM 3-morpholinopropanesulfonic acid (MOPS), 2 mM MgSO_4_ 7H_2_O, 0.1 mM CaCl_2_, 0.5 g/L NaCl, and 0.1 g/L yeast extract, pH 7.0) containing 2% (w/v) glucose and 1.0 mM IPTG for 72 h at 30°C (*n* = 3).

## Discussion

This study focused on establishment of an artificial pathway for biosynthesis of P(3HB-*co*-3HHx) copolymer from structurally unrelated and abundant glucose in non-PHA-producing *E. coli*. In total, three plasmids were constructed and introduced into *E. coli* for (*R*)-3HB-CoA formation and polymerization (*phaP*_D4N_ *C*_NSDG_ *J*_*Ac*_ and *phaAB1*_*Re*_), butyryl-CoA formation (*ccr*_*Me*_, *phaJ4a*_*Re*_, and *emd*_*Mm*_), and reverse β-oxidation (*bktB*_*Re*_, *had*_*Re*_, and *crt2*_*Re*_). The resulting recombinant strain harboring the 11 heterologous genes produced P(3HB-*co*-14 mol% 3HHx) with cellular content 41 wt% from glucose.

Lack of BktB_*Re*_ (entry 5 in Figure 2 and Supplementary Table S3) or Had_*Re*_-Crt2_*Re*_ (entry 6) in the pathway markedly reduced the 3HHx fraction within the synthesized PHA, demonstrating that (*R*)-3HHx-CoA monomer was formed through reverse β-oxidation from butyryl-CoA *via* (*S*)-3HHx-CoA (Figure 1). This was consistent with broad substrate specificities of BktB_*Re*_, Had_*Re*_, and Crt2_*Re*_ reported previously (Slater et al., 1998; Segawa et al., 2019). In the strain not harboring Had_*Re*_ *-*Crt2_*Re*_ (entry 6), PhaB1 is an enzyme potentially forming (*R*)-3HHx-CoA directly from 3-oxohexanoyl-CoA. Nevertheless, the low 3HHx fraction (1.2 mol%) within the polymer produced by the strain indicated little contribution of PhaB1 to provision of (*R*)-3HHx-CoA probably due to the low activity to the C_6_ substrate. It was also observed that PHA synthesis was severely impaired by deletion of *phaB1*_*Re*_ from pBKS-PCJAB (entry 4). In the engineered *R. eutropha* possessing multiple acetoacetyl-CoA reductase paralogs (Budde et al., 2010), the deletion of the major reductase gene *phaB1* was the important modification to achieve high 3HHx fraction in P(3HB-*co*-3HHx) synthesized from sugars (Insomphun et al., 2015; Zhang et al., 2019). This was due to change in metabolic flux distribution at the acetoacetyl-CoA node by the deletion of *phaB1*, decrease in (*R*)-3HB-CoA formation (mediated by minor reductase PhaB3), and consequent relative increase in crotonyl-CoA formation *via* (*S*)-3HB-CoA. In *E. coli* not possessing endogenous reductase, it was plausible that the lack of PhaB1 led to almost no formation of (*R*)-3HB-CoA from acetoacetyl-CoA. However, we initially supposed that the reverse β-oxidation mediated by Had_*Re*_ and Crt2_*Re*_ would be functional for conversion of acetoacetyl-CoA to crotonyl-CoA *via* (*S*)-3HB-CoA, which may lead to (*R*)-3HB-CoA formation by PhaJ_*Ac*_ and subsequent accumulation of P(3HB-co-3HHx) to some extent. The unexpectedly low-level PHA production by the *phaB1*-lacking strain indicated that the contribution of Had_*Re*_ and Crt2_*Re*_ to crotonyl-CoA formation appeared to be negligible in *E. coli* despite the actual activities to the corresponding C_4_ substrates in enzyme assay, namely, in the *phaB1* ^+^-strains, most crotonyl-CoA molecules were provided from (*R*)-3HB-CoA by dehydration catalyzed by PhaJ_*Ac*_ and then elongated to C_6_ intermediates.

Although the cellular content of P(3HB-*co*-14 mol% 3HHx) achieved in this study (41 wt%) was lower than 75 wt% of P(3HB-*co*-12 mol% 3HHx) observed in engineered *R. eutropha* (Zhang et al., 2019), the PHA production was comparable to each other (∼2.8 g/L). Wang et al (2015) reported P(3HB-*co*-3HHx) biosynthesis from glucose by recombinant *E. coli* and applied two pathways for (*R*)-3HHx-CoA formation from 3-oxohexanoyl-CoA: one is PhaB1-dependent reduction, and the other is FadB-dependent reverse β-oxidation *via* (*S*)-3HHx-CoA, and a combination of these two pathways resulted in the production of P(3HB-*co*-10 mol% 3HHx) with the cellular content of 12 wt% (0.65 g/L). Both present and previous studies demonstrated the higher 3HHx composition by the strains harboring reverse β-oxidation. The different reaction step between these studies was reduction of crotonyl-CoA to butyryl-CoA. We adopted a combination of NADPH-dependent crotonyl-CoA carboxylase/reductase (Ccr) and ethyl-malonyl-CoA decarboxylase (Emd) for generation of the important intermediate, butyryl-CoA, from crotonyl-CoA, while the previous pathway applied *trans*-enoyl-CoA reductase (Ter) with NADH dependency derived from *Treponema denticola*. The effects of catalytic properties including cofactor dependency on the copolyester biosynthesis are interesting points to be made clear. It is expected that further fine tuning of expression levels of the three modules (*phaPCJ-phaAB*, *ccr-phaJ4a-emd*, and *had-crt2-bktB*) by changing the plasmid backbone and promoters with an altered copy number and transcription strength, respectively, allows us to obtain strains with higher biosynthesis ability.

The effects of elimination of global factors on P(3HB-*co*-3HHx) biosynthesis in *E. coli* was also investigated in this study. Although it has been reported that inactivation of *cra*, *pdhR*, or *rng* promoted glucose utilization in *E. coli* (Ramseier et al., 1995; Saier and Ramseier, 1996; Sakai et al., 2007; and Maeda et al., 2017), these genetic modifications were not effective for the copolyester synthesis in *E. coli* BW25113. Interestingly, the ∆*rng* strain showed more accumulation of PHA with almost no 3HHx unit. As RNase G is an endonuclease degrading various mRNAs of glycolytic enzymes (Sakai et al., 2007), it was supposed that the deletion of *rng* resulted in enhancement of the Embden–Meyerhof pathway and relative decrease in carbon flux through the pentose-phosphate pathway. NADPH formed through the weakened pentose-phosphate pathway may be preferentially utilized by PhaB1 [apparent *K*_mNADPH_ = 19 μM (Haywood et al., 1988)] to form (*R*)-3HB-CoA, thus leading to reduced formation of butyryl-CoA by Ccr_*Me*_ [*K*_mNADPH_ = 250 μM (unpublished result)]. The block of acetate formation was a frequently applied strategy for enhancement of metabolic flux from acetyl-CoA to bioproducts (Krivoruchko et al., 2015; Matsumoto et al., 2017). It has been reported that the deletion of *pta* or deletion of *pta-ackA* and *poxB* increased production of P(3HB) homopolymer from glucose under microaerobic conditions (Kang et al., 2009; Wei et al., 2009). The present study showed that the single deletion of *pta* decreased the 3HHx composition, which may also be due to the decrease in the pentose-phosphate pathway flux caused by the modification. It should be noted that the PHA synthesis was remarkably impaired by the double deletion of *pta* and *poxB*, and pyruvate was significantly secreted instead of acetate. This suggested repression of conversion from pyruvate to acetyl-CoA by the lack of acetate formation under the aerobic condition.

We further examined introduction of a mutant of phosphite dehydrogenase capable of accepting NADP^+^ as the cofactor into the P(3HB-*co*-3HHx)-producing strain. The transformant produced the copolyester with not only higher content as previously observed for P(3HB) synthesis (Miyahara et al., 2018) but also higher 3HHx composition by the two-stage cultivation with phosphite than that produced in the absence of phosphite (Table 2). This was consistent with more efficient formation of (*R*)-3HB-CoA by PhaB1 as well as butyryl-CoA by the Ccr-Emd combination owing to the phosphite-dependent regeneration of NADPH. The introduction of phosphite oxidation accompanied with a slight decrease of glucose consumption and the resulting increase in the PHA yield to glucose, which might be due to repression of glucose degradation responding to the NADPH regeneration associated with the phosphite oxidation. Taken together, this study demonstrated the usefulness of *E. coli* for the production of PHA copolyesters from structurally unrelated carbon sources, and further modifications are expected to improve the productivity.

## Data Availability

The original contributions presented in the study are included in the article/Supplementary Material; further inquiries can be directed to the corresponding author.
