# Supplementary material for: Biosynthesis of Poly(3-hydroxybutyrate-co-3-hydroxyhexanoate) From Glucose by Escherichia coli Through Butyryl-CoA Formation Driven by Ccr-Emd Combination
Source: Front Bioeng Biotechnol. 2022 May 12;10:888973. doi: 10.3389/fbioe.2022.888973 (PMC9134075; doi:10.3389/fbioe.2022.888973)
Supplement: Supplementary file 1 [file DataSheet1.PDF]

Supporting Information

Biosynthesis of poly(3-hydroxybutyrate-*co*-3-hydroxyhexanoate) from glucose by *Escherichia coli* through butyryl-CoA formation driven by Ccr-Emd combination

Shu Saito[1], Ryu Imai[1], Yuki Miyahara[2], Mari Nakagawa[1], Izumi Orita[1],
Takeharu Tsuge[2], and Toshiaki Fukui[1,*]

1) School of Life Science and Technology, 2) School of Materials and Chemical Technology, Tokyo Institute of Technology, Yokohama, Japan

*Corresponding author
Toshiaki Fukui
School of Life Science and Technology
Tokyo Institute of Technology
Tel/Fax: +81-45-924-5766
e-mail: tfukui@bio.titech.ac.jp

Table S1
Table S2
Table S3
Table S4
Table S5

Supplementary Table S1. Strains and plasmids used in this study.

Strain or plasmid	Relevant marker	Source or reference
Escherichia coli		
DH5*α*	F^{-}, *φ*80*lacZ*ΔM15, Δ(*lacZYA*-*argF*) U169, *deoR*, *recA*1, *endA*1, *hsdR*17(r_K^{-}, m_K^{+}), *phoA*, *supE*44, λ^{-}, *thi*-1, *gyrA*96, *relA*1	Lab stock
JM109	*endA*1, *recA*1, *gyrA*96, *thi*-1, *hsdR*17(r_K^{-} m_K^{+}), *relA*1, *supE*44, Δ(*lac*-*proAB*), F'[*traD*36, *proAB*, $lacI^q$, *lacZ*ΔM15]	Lab stock
BW25113	*rrnB*, Δ*lacZ*4787, *hsdR*514, Δ*araBAD*567, Δ*rhaBAD568*, *rph*-1	Datsenko and Wanner (2000)
JWΔcra	JW0078-KC (BW25113 Δ*cra*::Km^r) derivative, Δ*cra*::FRT	This study
JWΔpdhR	JW0109-KC (BW25113 Δ*pdhR*::Km^r) derivative, Δ*pdhR*::FRT	This study
JWΔrng	JW3216-KC (BW25113 Δ*rng*::Km^r) derivative, Δ*rng*::FRT	This study
JWΔcraΔrng	JWΔcra derivative, Δ*rng*::FRT	This study
JWΔpgi	JW3985-KC (BW25113 Δ*pgi*::Km^r) derivative, Δ*pgi*::FRT	This study
JWΔpgiΔrng	JWΔpgi derivative, Δ*rng*::FRT	This study
JWΔpta	JW2294-KC (BW25113 Δ*pta*::Km^r) derivative, Δ*pta*::FRT	This study
JWΔptaΔpoxB	JWΔpta derivative, Δ*poxB*::FRT	This study
**Plasmids**		
pBKS-PCJAB	pBluescript II KS(+) derivative; P_{lac}-P_{Ac}-$phaP_{\mathrm{D4N}}C_{\mathrm{NSDG}}J_{Ac}$, P_{Re}-$phaAB1_{Re}$	This study
pBKS-PCJA	pBluescript II KS(+) derivative; P_{lac}-P_{Ac}-$phaP_{\mathrm{D4N}}C_{\mathrm{NSDG}}J_{Ac}$, P_{Re}-$phaA_{Re}$	This study
pBBRtac	pBBR1-MCS2 derivative; P_{tac}	Fukui et al. 2009
pBtac-CJ$_{\mathrm{Re}}$E	pBBRtac derivative, P_{tac}-ccr_{Me}-$phaJ4a_{Re}$-emd_{Mm}	This study
pBtac-CJ$_{\mathrm{Re}}$EB	pBBRtac derivative, P_{tac}-ccr_{Me}-$phaJ4a_{Re}$-emd_{Mm}-$bktB_{Re}$	This study
pBtac-CJ4$_{\mathrm{Pa}}$E	pBBRtac derivative, P_{tac}-ccr_{Me}-$phaJ4_{Pa}$-emd_{Mm}-$bktB_{Re}$	This study
pSTV-HC	pSTV28 derivative; P_{tac}-had_{Re}-$crt2_{Re}$	This study
pSTV-HCB	pSTV28 derivative; P_{tac}-had_{Re}-$crt2_{Re}$-$bktB_{Re}$	This study
pSTV-PCB	pSTV28 derivative; P_{tac}-$paaH1_{Re}$-$crt2_{Re}$-$bktB_{Re}$	This study
pMW-Gm-ptxD$_{\mathrm{EAAR}}$ABC	pMW218 derivative, ΔKm^r::Gm^r, $ptxD_{\mathrm{EAAR}}$-*ptxABC*	This study

FRT, FLP recombinase target; *Ac*, *Aeromonas caviae*; *Me*, *Methylorubrum extorquens*; *Mm*, *Mus musculus*; *Re*, *Ralstonia eutropha*.

Supplementary Table S2. The sequences of primers used in this study.

Primer	Sequence (5'-3')	Note
Construction of plasmids		
del-phaB1-inv5	CCTGCCGGCCTGGTTCAACCAGTCG	For construction of pBKS-PCJA
del-phaB1-inv3	GTCCACTCCTTGATTGGCTTCGTTA	
del-PphaP-inv5	AAAGAGGAGAAATTAACTATGGCTGCAAGCGCAGCACCGGCCT	For construction of pBtac-CJ$_{Re}$E
del-PphaP-inv3	GGGGGTGGGCGAAGAACTCCAGCAT	
tac-5	TTGAATTCGAGCTCAATATTCTGAAATGAGCTGTTGA	
tac-Rv	GGTTCAATTCTGTTTCCTGTG	
bktB-Fw1	CGCTTCGAATCTAGAAAGGAGGC	For construction of pBtac-CJ$_{Re}$EB
bktB-Rv1	ATCCACCCCTTCCTCAGATACGC	
phaJ4a-Inv1	GTCGATAGTCTCCTCTTGACGATAAAGC	For construction of pBtac-CJ4$_{Pa}$E
phaJ4a-Inv2	GGATCCGTTTTTTTGGGCTAGCAGGAGGA	
phaJ4Pa_N	ATGCCATTCGTACCCGTAGCA	
phaJ4Pa_C	TCAGACGAAGCAGAGGCTGA	
A0602-F	ATGCAAATCCAAGGCAACGTATTCA	For construction of pSTV-HC
A0602-R-Fus	ATGTATTTGCCTTTACTTGGGCTGCATCCGGA	
A3307-R	GCCTTAGCGATGCTGGAAATT	
A3307-F-Fus	CCAAGTAAAGGCAAATACATAGGAGAAGACA	
pSTV-inv5	TATCGATGATAAGCTGTCAAACA	
pSTV-inv3	CATTAATGAATCGGCCAACGCGC	
pSTV-inv5_2	GGCTTAAGCCAGCCCCGACACC	For construction of pSTV-HCB
pSTV-inv3_2	TTAGCGATGCTGGAAATTCGGG	
Ptac-rrnB-Inv3	ATGATAATCCTCCTGAATTCAATTGTTATCCGCTCACAATTCCACACATT	For construction of pSTV-PCB
pSTV-AA-Inv_3307N	GGCAAATACATAGGAGAAGACAT	
A0282_N	ATGAGCATCAGGACAGTGGGCA	
A0282_C	TTACTTGCTATAGACGTACACG	
Construction of double-knockout strains of *E. coli*		
rng-KC-Fw	TGAATTATTAAGTAATTTTAACGCACTGCG	For construction of JWΔcraΔrng and JWΔpgiΔrng
rng-KC-Rv	CCTTTGCCGGATGGCGGCCCAGCATCTG	
poxB-KC-Fw	CCTTATTATGACGGGAAATGCCACCC	For construction of JWΔptaΔpoxB
poxB-KC-Rv	GATGAACTAAACTTGTTACCGTTATC	

Supplementary Table S3. P(3HB-*co*-3HHx) biosynthesis from glucose by *E. coli* JM109-derived recombinant strains.

Entry	Plasmid 1	Plasmid 2	Plasmid 3	Dry cell mass (g/L)	PHA [g/L]	Residual cell mass (g/L)	PHA content [wt%]	3HHx [mol%]	Monomer amount in PHA [mmol/L-culture]	
									3HB	3HHx
1	pBKS-PCJAB			9.86 ±0.05	5.64 ±0.18	4.22 ±0.19	57.2 ±1.8	0	65.6 ±2.0	0
2	pBKS-PCJAB	pBtac-CJ$_{Re}$E		6.29 ±0.59	2.37 ±0.41	3.92 ±0.27	37.5 ±3.4	0	27.6 ±4.8	0
3	pBKS-PCJAB	pBtac-CJ$_{Re}$E	pSTV-HCB	6.87 ±0.26	2.82 ±0.30	4.05 ±0.05	41.0 ±2.8	14.0 ±0.3	27.0 ±2.9	4.4 ±0.5
4	pBKS-PCJA	pBtac-CJ$_{Re}$E	pSTV-HCB	3.51 ±0.04	0.14 ±0.02	3.38 ±0.03	3.9 ±0.52	21.5 ±0.8	1.2 ±0.2	0.32 ±0.05
5	pBKS-PCJAB	pBtac-CJ$_{Re}$E	pSTV-HC	4.49 ±0.05	0.59 ±0.08	3.91 ±0.12	13.0 ±1.8	0	6.8 ±0.9	0
6	pBKS-PCJAB	pBtac-CJ$_{Re}$EB		8.06 ±0.15	3.39 ±0.13	4.66 ±0.17	42.1 ±1.7	1.2 ±0.1	38.8 ±1.4	0.49 ±0.06

The cells were cultivated in a 100 ml LB medium containing 2% (w/v) glucose and 1 mM IPTG for 48 h at 30˚C (*n*=3).

pBSKS-PCJAB ($phaP_{\mathrm{D4N}}C_{\mathrm{NSDG}}J_{Ac}$-$phaAB_{Re}$), pBKS-PCJA ($phaP_{\mathrm{D4N}}C_{\mathrm{NSDG}}J_{Ac}$-$phaA_{Re}$)

pBtac-CJ$_{Re}$E (ccr_{Me}-$phaJ4a_{Re}$-emd_{Mm}), pBtac-CJ$_{Re}$EB (ccr_{Me}-$phaJ4a_{Re}$-emd_{Mm}-$bktB_{Re}$)

pSTV-HCB (had_{Re}-$crt2_{Re}$-$bktB_{Re}$), pSTV-HC, (had_{Re}-$crt2_{Re}$)

Supplementary Table S4. Effects of replacements of (*R*)-hydratase gene and/or 3HB-CoA dehydrogenase gene on P(3HB-*co*-3HHx) biosynthesis from glucose by *E. coli* JM109-derived recombinant strains.

Plasmid 1	Plasmid 2 (*R*-hydratase gene)	Plasmid 3 (3HB-CoA DH gene)	Dry cell mass (g/L)	PHA [g/L]	Residual cell mass (g/L)	PHA content [wt%]	3HHx comp. [mol%]	Monomer amount in PHA [mmol/L-culture]	
								3HB	3HHx
pBKS-PCJAB	pBtac-CJ$_{\mathrm{Re}}$E ($phaJ4a_{Re}$)	pSTV-HCB (had_{Re})	6.55 ±0.18	2.06 ±0.19	4.48 ±0.04	31.5 ±2.0	15.4 ±1.5	19.4 ±1.4	3.5 ±0.1
		pSTV-PCB ($paaH1_{Re}$)	6.68 ±0.13	2.05 ±0.14	4.63 ±0.05	30.7 ±1.5	14.2 ±0.4	19.6 ±1.4	3.2 ±0.2
	pBtac-CJ4$_{\mathrm{Pa}}$E ($phaJ4_{Pa}$)	pSTV-HCB (had_{Re})	6.58 ±0.04	1.93 ±0.03	4.66 ±0.06	29.3 ±0.5	16.1 ±0.2	17.9 ±0.3	3.4 ±0.04
		pSTV-PCB ($paaH1_{Re}$)	6.78 ±0.05	2.09 ±0.22	4.69 ±0.21	30.8 ±3.2	14.3 ±1.0	19.9 ±1.8	3.3 ±0.6

The cells were cultivated in a 100 ml LB medium containing 2% (w/v) glucose and 1 mM IPTG for 48 h at 30˚C (*n*=3).

pBKS-PCJAB ($phaP_{\mathrm{D4N}}C_{\mathrm{NSDG}}J_{Ac}$-$phaAB_{Re}$)

pBtac-CJ$_{\mathrm{Re}}$E (ccr_{Me}-$phaJ4a_{Re}$-emd_{Mm}), pBtac-CJ4$_{\mathrm{Pa}}$EB (ccr_{Me}-$phaJ4_{Pa}$-emd_{Mm}-$bktB_{Re}$)

pSTV-HCB (had_{Re}-$crt2_{Re}$-$bktB_{Re}$), pSTV-PCB ($paaH1_{Re}$-$crt2_{Re}$-$bktB_{Re}$)

Supplementary Table S5. Effects of mutation(s) in sugar metabolism-regulating genes and acetate formation on P(3HB-*co*-3HHx) biosynthesis from glucose by *E. coli* BW25113-derived recombinant strains harboring pBKS-PCJAB/pBtac-cJ$_{Re}$e/pSTV-HCB.

Entry	Host strain	Dry cell mass (g/L)	PHA [g/L]	Residual cell mass (g/L)	PHA content [wt%]	3HHx comp. [mol%]	Monomer amount in PHA [mmol/L-culture]		Glucose consumption [g/L]	Acetate formation [g/L]
							3HB	3HHx		
7	BW25113 (control)	6.10 ±0.17	1.97 ±0.14	4.13 ±0.03	32.2 ±1.4	15.7 ±0.24	18.3 ±1.4	3.42 ±0.21	17.0 ±0.5	0.73 ±0.11
Sugar metabolisms										
8	JWΔcra	1.60 ±0.01	0.07 ±0.00	1.53 ±0.01	4.5 ±0.1	3.1 ±0.2	0.80 ±0.02	0.03 ±0.01	6.8 ±0.5	2.1 ±0.2
9	JWΔpdhR	3.02 ±0.18	0.36 ±0.01	2.66 ±0.17	11.8 ±0.4	trace	4.1 ±0.1	trace	9.8 ±0.3	0.56 ±0.14
10	JWΔrng	8.45 ±0.24	3.63 ±0.33	4.82 ±0.13	42.9 ±2.8	trace	42.2 ±3.9	trace	19.9 ±0.1	0.17 ±0.02
11	JWΔcraΔrng	8.23 ±0.12	3.28 ±0.08	4.95 ±0.08	39.8 ±0.6	trace	38.1 ±0.9	trace	19.9 ±0.1	0.16 ±0.02
12	JWΔpgi	4.01 ±0.18	0.75 ±0.06	3.27 ±0.12	18.6 ±0.7	22.2 ±1.1	6.3 ±0.4	1.8 ±0.2	12.9 ±0.6	1.3 ±0.1
13	JWΔpgiΔrng	3.82 ±0.06	0.66 ±0.05	3.10 ±0.02	18.8 ±0.7	6.5 ±0.1	7.6 ±0.5	0.54 ±0.03	11.2 ±0.2	1.2 ±0.1
Acetate formation										
14	JWΔpta	5.29 ±0.09	2.19 ±0.06	3.10 ±0.03	41.4 ±0.5	1.9 ±0.2	24.9 ±0.6	0.47 ±0.1	14.2 ±0.3	0.51 ±0.03
15	JWΔptaΔpoxB	1.65 ±0.12	trace	1.65 ±0.12	trace	0	trace	0	6.9 ±0.1	1.4 ±0.1 (pyruvate)

The cells were cultivated in a 100 ml LB medium containing 2% (w/v) glucose and 1mM IPTG for 48 h at 30˚C (*n*=3).

pBKS-PCJAB (*phaP*$_{D4N}$*C*$_{NSDG}$*J*$_{Ac}$-*phaAB*$_{Re}$), pBtac-CJ$_{Re}$E (*ccr*$_{Me}$-*phaJ4a*$_{Re}$-*emd*$_{Mm}$), pSTV-HCB (*had*$_{Re}$-*crt2*$_{Re}$-*bktB*$_{Re}$)
